# Identification of Human GnIH Homologs, RFRP-1 and RFRP-3, and the Cognate Receptor, GPR147 in the Human Hypothalamic Pituitary Axis

**DOI:** 10.1371/journal.pone.0008400

**Published:** 2009-12-22

**Authors:** Takayoshi Ubuka, Kevin Morgan, Adam J. Pawson, Tomohiro Osugi, Vishwajit S. Chowdhury, Hiroyuki Minakata, Kazuyoshi Tsutsui, Robert P. Millar, George E. Bentley

**Affiliations:** 1 Department of Integrative Biology and Helen Wills Neuroscience Institute, University of California, Berkeley, California, United States of America; 2 Medical Research Council Human Reproductive Sciences Unit, Centre for Reproductive Biology, The Queen's Medical Research Institute, Edinburgh, United Kingdom; 3 Department of Biology, Waseda University, Tokyo, Japan; 4 Suntory Institute for Bioorganic Research, Osaka, Japan; 5 Department of Medical Biochemistry, University of Cape Town, Cape Town, South Africa; New Mexico State University, United States of America

## Abstract

The existence of a hypothalamic gonadotropin-inhibiting system has been elusive. A neuropeptide named gonadotropin-inhibitory hormone (GnIH, SIKPSAYLPLRF-NH_2_) which directly inhibits gonadotropin synthesis and release from the pituitary was recently identified in quail hypothalamus. Here we identify GnIH homologs in the human hypothalamus and characterize their distribution and biological activity. GnIH homologs were isolated from the human hypothalamus by immunoaffinity purification, and then identified as MPHSFANLPLRF-NH_2_ (human RFRP-1) and VPNLPQRF-NH_2_ (human RFRP-3) by mass spectrometry. Immunocytochemistry revealed GnIH-immunoreactive neuronal cell bodies in the dorsomedial region of the hypothalamus with axonal projections to GnRH neurons in the preoptic area as well as to the median eminence. RT-PCR and subsequent DNA sequencing of the PCR products identified human GnIH receptor (GPR147) mRNA expression in the hypothalamus as well as in the pituitary. *In situ* hybridization further identified the expression of GPR147 mRNA in luteinizing hormone producing cells (gonadotropes). Human RFRP-3 has recently been shown to be a potent inhibitor of gonadotropin secretion in cultured sheep pituitary cells by inhibiting Ca^2+^ mobilization. It also directly modulates GnRH neuron firing. The identification of two forms of GnIH (RFRP-1 and RFRP-3) in the human hypothalamus which targets human GnRH neurons and gonadotropes and potently inhibit gonadotropin in sheep models provides a new paradigm for the regulation of hypothalamic-pituitary-gonadal axis in man and a novel means for manipulating reproductive functions.

## Introduction

Gonadotropin-releasing hormone (GnRH) is the primary stimulator of gonadotropin secretion [1]–[5]. A neuropeptide inhibitor of gonadotropin secretion has also been postulated [6]–[8]. The recent identification of an avian hypothalamic dodecapeptide that inhibits pituitary gonadotropin release implies that such a factor might exist in vertebrates [9]. This factor, named gonadotropin-inhibitory hormone (GnIH), is synthesized in neurons of the paraventricular nucleus (PVN) in birds [9]–[13]. The GnIH neurons project to the median eminence, providing neuroanatomical infrastructure to allow secretion into the hypophysial portal system and thus regulate pituitary function [9]–[13]. The cognate G protein-coupled receptor (GPCR) for GnIH was also identified in the quail pituitary [14] and GnIH was shown to act on the pituitary to suppress synthesis and release of gonadotropins [15]. Accordingly, GnIH inhibits the development and maintenance of gonadotropin-dependent gonadal functions [16]. Thus, while GnIH serves an important physiological role in birds [9]–[19], there has been limited evidence that the same is true for mammals.

GnIH homologs are present in the brains of non-human vertebrates, including mammals, amphibians and fish [20], [21]. These peptides belong to the RFamide-related peptide (RFRP) [22] family and possess a characteristic C-terminal LPXRFamide (X = L or Q) motif [20], [21]. The receptors for GnIH homologs have also been characterized in vertebrates [14], [20]–[22]. Quail GnIH and rat GnIH peptide homolog, RFRP-3, inhibits luteinizing hormone (LH) secretion in Syrian hamsters [23] and rats [24], [25] *in vivo*. It was recently shown that RFRP-3 inhibits pulsatile gonadotropin release *in vivo* as well as from cultured pituitary cells in sheep [26], [27] and cattle [28], suggesting that a hypothalamic gonadotropin-inhibitory system also exists in mammals. Accordingly, a GnIH/RFRP system might be a conserved property of vertebrates. However, these mammalian GnIH peptide homologs were inferred from genomic sequences and the processed peptides have yet to be unequivocally identified.

Here we first analyzed the existence of GnIH-immunoreactive (-ir) material in human hypothalamus by immunocytochemistry (ICC). We further investigated whether there were interactions of GnIH-ir neurons with GnRH neurons by double-label ICC. We then isolated human GnIH peptide homologs (human RFRP-1 and RFRP-3) by immunoaffinity purification and identified the structure of the peptides by mass spectrometry. A G-protein coupled receptor, GPR147 (OT7T022) has been identified as the cognate receptor for RFRPs in studies investigating the role of these peptides in the central nervous system [29]. Accordingly GPR147 mRNA expression was analyzed in the hypothalamus and the pituitary by RT-PCR and DNA sequencing of the PCR products. *In situ* hybridization further revealed the expression of GPR147 mRNA in pituitary cells including luteinizing hormone (LH) producing cells. The human RFRP-3 was recently shown to potently inhibit GnRH stimulation of gonadotropin secretion from sheep *in vivo* and from cultured gonadotropes through inhibition of Ca^2+^ mobilization [26]. The identification of RFRP-1 and RFRP-3 and the cognate receptor GPR147 along with the demonstration of gonadotropin inhibition prompts revision of our understanding of the central control mechanism of human reproduction.

## Results

### Localization of GnIH-ir neurons in the human hypothalamus

**Figure 1:**
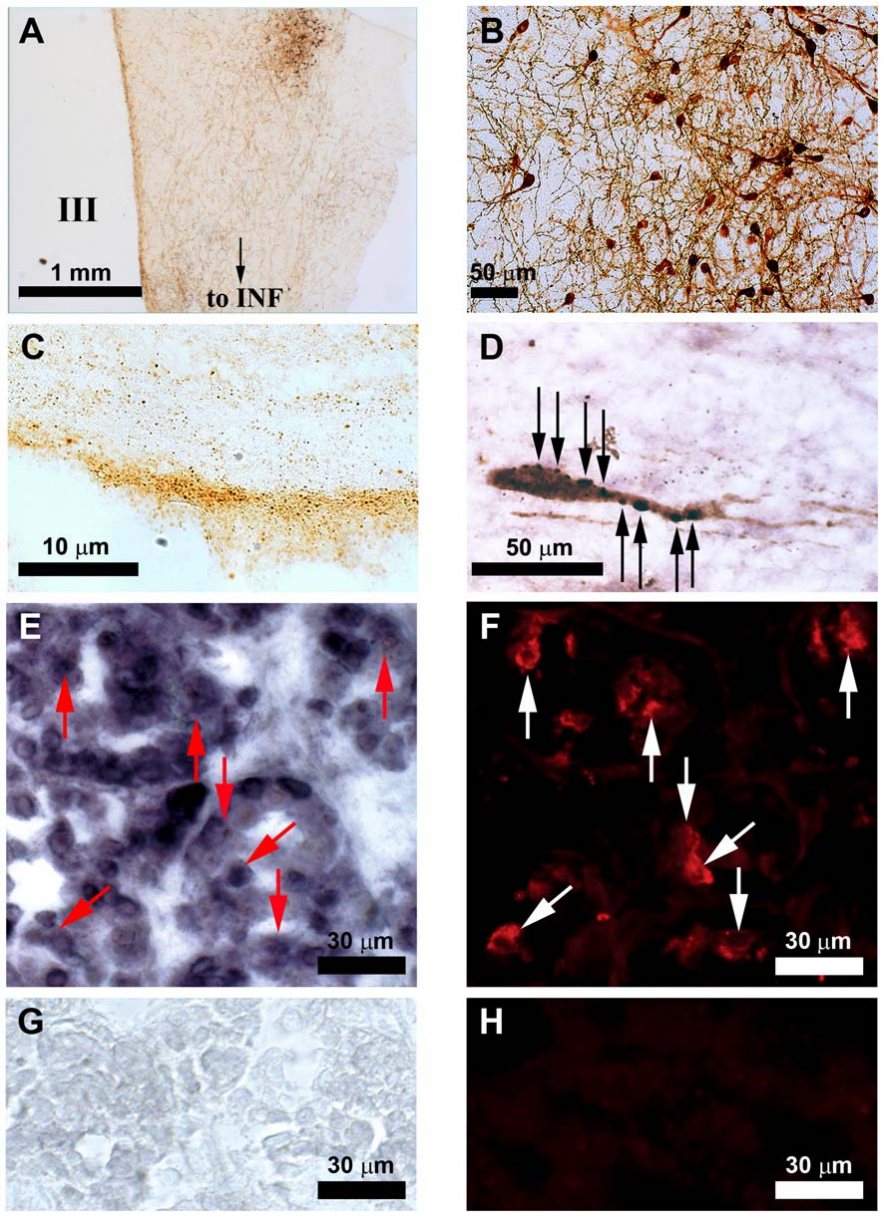
GnIH immunoreactive neurons in the human hypothalamus and GnIH receptor mRNA in the human pituitary. (A) Coronal section of adult human hypothalamus showing GnIH immunoreactive (-ir) neurons clustered in the dorsomedial region of the hypothalamus with their fibers extending to the infundibulum (INF). III, third ventricle. Bar, 1 mm. (**B**) Higher magnification of GnIH-ir neuronal cell bodies. Bar, 50 µm. (**C**) Sagittal section of the median eminence showing dense population of GnIH-ir fibers within the external layer. Bar, 10 µm. (**D**) GnIH-ir axon terminal like structures (stained in purple, indicated by black arrows) in close proximity to a GnRH-ir neuronal cell body (stained in brown) in the preoptic area. Bar, 50 µm. (**E and F**) *In situ* hybridization of human GnIH receptor (GPR147) mRNA in the human anterior pituitary (**E**) in combination with immunocytochemistry for luteinizing hormone (LH) (**F**). Red arrows in (**E**) and white arrows in (**F**) in equivalent positions show cells which express both GPR147 mRNA and LH. Bars, 30 µm. (**G and H**) *In situ* hybridization using sense RNA probe (**G**) and immunocytochemistry without LH primary antiserum (**H**) served as controls. Bars, 30 µm.

ICC using avian GnIH antibody identified a group of GnIH-ir neurons in the dorsomedial region of the human hypothalamus (Fig. 1A, 1B). Some GnIH-ir fibers emanated from the infundibulum of the hypothalamus (Fig. 1A) and terminated in the external layer of the median eminence (Fig. 1C). GnIH-ir neuronal axon terminal-like structures were further observed on GnRH neurons in the preoptic area (Fig. 1D).

### Identification of mature human GnIH peptide homologs

**Figure 2:**
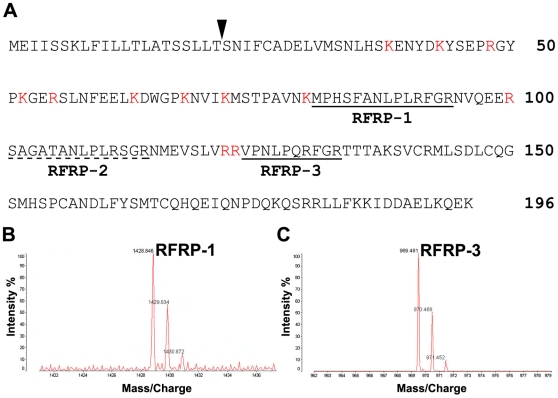
Identification of human GnIH homologs. (**A**) Amino acid sequence of the human GnIH precursor polypeptide. The arrowhead shows the predicted cleavage site of the N-terminal secretory signal peptide [29]. Two peptide sequences which have LPXRF (X = L or Q) sequence at their C-termini with glycine as an amidation signal and arginine as an endoproteolytic basic amino acid are underlined (RFRP-1, RFRP-3). One peptide sequence which has LPLRS sequence at its C-terminus with glycine and arginine is underlined with a broken line (RFRP-2). The basic amino acids which could be proteolytic cleavage sites are shown in red. (**B**) Chromatogram of MALDI-TOF MS of the native human RFRP-1. (**C**) Chromatogram of MALDI-TOF MS of the native human RFRP-3.

We identified the fully processed forms of GnIH homologs in the extract of human hypothalamus by using an avian GnIH antibody-conjugated immunoaffinity column and mass spectrometry. The human genome database revealed that a putative homolog of the GnIH precursor polypeptide gene is present in humans (GenBank NM_022150; 29), but the processed peptide products had not been identified until now. Because of several potential cleavage sites in the putative GnIH precursor polypeptide (Fig. 2A), there were various possible GnIH peptide products. Thus, based on the human genome, putative human RFRP-1 could be 12, 20, 24, 29, 37, 40, 44, 49 or 54 amino acids in length and human RFRP-3 could be 8 or 31 amino acids in length (Fig. 2A). Hypothalamic tissue from adult men and women was used to purify and identify human RFRP peptides by immunoaffinity purification followed by MALDI-TOF MS. GnIH-ir material in the extract of human hypothalamus showed a molecular ion peak of 1428.85 m/z ([M+H]^+^) (Fig. 2B). This value was in accord with the theoretical mass of the predicted human RFRP-1 (MPHSFANLPLRF-NH_2_) [1428.75 m/z ([M+H]^+^)]. GnIH antiserum isolated another immunoreactive material in the hypothalamic extracts with a molecular ion peak of 969.48 m/z ([M+H]^+^) (Fig. 2C). This value was in accord with the theoretical mass of the predicted human RFRP-3 (VPNLPQRF-NH_2_) [969.56 m/z ([M+H]^+^)]. To confirm the data obtained by MALDI-TOF MS analyses of the endogenous GnIH-ir material, peptides having the suggested sequences were synthesized and compared with the native peptides for retention time on reverse-phase high performance liquid chromatography (HPLC) and mass number. Both native and synthetic peptides showed a similar retention time using HPLC and a similar molecular mass [RFRP-1: 1428.91 m/z ([M+H]^+^), RFRP-3: 969.81 m/z ([M+H]^+^)] (Table 1). Furthermore, fragmentation of the synthetic and native peptides by tandem MS confirmed the structures of human RFRP-1 and RFRP-3.

**Table 1:** Behavior of Native and Synthetic Human RFRP-1 and RFRP-3 on MALDI-TOF MS.

	Observed Mass m/z ([M+H]^+^)	Observed Mass m/z ([M+H]^+^)	Theoretical Mass m/z ([M+H]^+^)
	Native	Synthetic	
Human RFRP-1	1428.85	1428.91	1428.75
Human RFRP-3	969.48	969.81	969.56

### Expression of GPR147 mRNA in the human hypothalamus and in the pituitary

**Figure 3:**
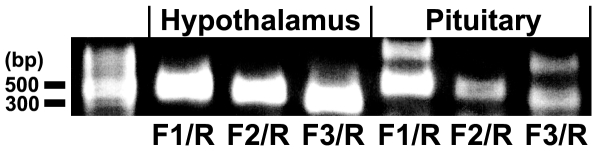
Gel image of RT-PCR products of human GnIH receptor (GPR147) mRNA. cDNAs corresponding to 1 µg total RNA extracted from human hypothalamus or pituitary were used for a PCR reaction. Combinations of forward primers (F1, F2 and F3) with a reverse primer (R) produced RT-PCR products of 479, 415 and 325 bp using hypothalamic and pituitary cDNAs as the template. The PCR products were sequenced and confirmed to be derived from GPR147 mRNA (NM_022146). F1/R and F3/R primer combinations produced additional RT-PCR products in the pituitary although the subsequent DNA sequencing identified that they were derived from *Homo sapiens* RAB11 family interacting protein 1 and NEDD4 binding protein 1 mRNAs, respectively.

The expression of human RFRP receptor, GPR147, mRNA was analyzed in the human hypothalamus as well as in the pituitary. Combinations of various forward primers (F1, F2 and F3) and a reverse primer (R) produced expected RT-PCR products of 479, 415 and 325 bp in the hypothalamus (Fig. 3). The PCR products were sequenced and confirmed to be derived from GPR147 mRNA (GenBank NM_022146). RT-PCR and subsequent DNA sequencing of the PCR products also identified the same 479, 415 and 325 bp GPR147 cDNAs expressed in the pituitary. F1/R and F3/R primer combinations produced additional RT-PCR products in the pituitary; DNA sequencing identified that they were derived from *Homo sapiens* RAB11 family interacting protein 1 and NEDD4 binding protein 1 mRNAs, respectively. *In situ* hybridization using an antisense RNA probe for GPR147 mRNA in conjunction with ICC for human LH showed the expression of GPR147 mRNA in the gonadotrope of the adult human anterior pituitary (Fig. 1, E and F). *In situ* hybridization using sense RNA probe (Fig. 1G) and immunocytochemistry without LH primary antiserum (Fig. 1H) served as controls.

## Discussion

We identified the fully processed forms of GnIH homologs in an extract of human hypothalamus by using an avian GnIH antibody-conjugated immunoaffinity column and mass spectrometry. Two GnIH homologs were identified, the structures of which were MPHSFANLPLRF-NH_2_ (human RFRP-1) and VPNLPQRF-NH_2_ (human RFRP-3). The human genome database also predicted the presence of an RFRP-like peptide which has a C-terminus LPLRSamide motif, but this was not detected, possibly because of the difference in the C-terminal amino acid compared to avian GnIH, or because this peptide is not processed from the precursor. The precise mechanism of how the GnIH/RFRP precursor protein is processed into mature peptides and how this process differs between species is unknown.

GnIH-ir neurons were observed in the dorsomedial region of the human hypothalamus. The location of GnIH neuronal cell bodies were in general agreement to hamsters (dorsomedial hypothalamus; 23) and sheep (dorsomedial hypothalamic nucleus and paraventricular nucleus; 26). GnIH-ir axon terminal like structures were observed in close proximity to GnRH neurons in the preoptic area suggesting the regulation of GnRH neurons by GnIH. The interaction of GnIH-ir fibers with GnRH neurons is consistent with results in birds [17], [19], rodents [23] sheep [31] and monkey [32]. In starlings, GnIH receptor (GPR147) mRNA is expressed in GnRH neurons [19] and we demonstrated expression of the GPR147 in human hypothalamus. The possibility that GnIH affects GnRH neuron activity is supported by the recent demonstration that RFRP administration reduces the firing activity [33], [34] and immediate early gene expression in GnRH neurons [35]. GnIH-ir axons also projected to the neurosecretory zone of the median eminence suggesting that GnIH might also directly regulate pituitary function. The expression of the human GPR147 mRNA in the pituitary supports this notion. Moreover, *in situ* hybridization for GPR147 mRNA in conjunction with immunocytochemistry for human LH further showed the expression of GPR147 mRNA in gonadotropes of the adult human anterior pituitary. Accordingly, human GnIH homologs may regulate gonadotropin secretion by inhibiting GnRH neurons as well as directly acting on gonadotrope cells in the pituitary as was clearly demonstrated in isolated pituitary cells in the sheep [26], [27].

Our identification of RFRP-1 and RFRP-3 peptides in the human hypothalamus and the ICC demonstration of targeting GnRH neurons in the human hypothalamus along with the expression of GPR147 in human gonadotropes provide a cogent framework for RFRP regulation of human reproduction. The question arises as to whether the human RPRP peptides are biologically active and inhibit gonadotropins. Similarly, we studied the effects of human RFRP on the mouse gonadotrope LβT2 cell line but detected no effects on any signaling pathway. When we checked GPR147 expression by RT-PCR no products were detected in this immature gonadotrope cell line (data not shown). We detected inhibitory effects in human fetal pituitary cells, but these were inconsistent even though they exhibited robust gonadotropin responses to GnRH (data not shown). This variability may be due to the immaturity of the gonadotropes. However, human RFRP-3, which is identical in sequence to sheep RFRP-3, potently inhibits gonadotropin secretion *in vivo* in sheep and GnRH stimulation of gonadotropin secretion from cultured sheep pituitary cells [26], [27]. This strongly suggests RFRP-3 would also be active in the direct inhibition of gonadotropin release from gonadotropes in man. The demonstration that RFRP regulates GnRH neuron firing and early gene expression [33]–[35] suggests that the peptides may also be effective at this level in man. The question also arises as to whether RFRP-1 and RFRP-3 have similar or different functions. It appears from the large pain literature that they both bind GPR147 with similar affinity and recruit similar signalling pathways. Thus, the presence of two peptide products from the same gene may be a means for signal amplification.

The identification of novel RFamide-related peptides (RFRP-1 and RFRP-3) in the human hypothalamus and the demonstration that human RFRP-3 has potent inhibitory actions on gonadotropin secretion in animal models opens up new vistas for understanding the regulation of reproduction. Many factors such as stress, anorexia, diabetes, obesity and photoperiod inhibit gonadotropin secretion. The possible role of GnIH in mediating these effects has been implicated in animal models [30], [36], [37]. GnRH analogues are extensively employed to manipulate the hypothalamic-pituitary-gonadal axis in various clinical conditions [38]–[40]. In view of its potent inhibition of gonadotropes, GnIH has the potential of an alternative or adjunct therapeutic agent to inhibit gonadotropins and steroid hormones. Thus this endogenous inhibitor of gonadotropin secretion has therapeutic potential in the treatment of hormone-dependent diseases such as precocious puberty, endometriosis, uterine fibroids, benign prostatic hyperplasia and prostatic and breast cancers. Human GnIH may also have potential as a novel contraceptive.

## Materials and Methods

### Human hypothalamus and pituitary tissue

Frozen and fixed adult human hypothalamus and pituitary tissue were obtained from Harvard Brain Tissue Resource Center (Belmont, MA). All donations were with fully informed consent by written statements from the deceased individuals. The research was approved by the ethics committee of the Harvard Brain Tissue Resource Center, McLean Hospital (Belmont, MA) (Director: Francine M. Benes, M.D., Ph.D.) and the IRB. All procedures were performed in accordance with the NIH Guide and under an approved protocol from the University of California.

### Immunocytochemistry of human GnIH peptide

Immunocytochemical analysis of GnIH was conducted with slight modifications of our previous method [19] on coronal sections at 40 µm thickness of five fixed human hypothalami. Sections were first post-fixed in 4% PFA for 30 min, and incubated in 0.3% H_2_O_2_ in absolute methanol for 20 min to suppress endogenous peroxidase activity after washing the sections three times in phosphate-buffered saline (PBS; 10 mM phosphate buffer, 0.14 M NaCl, pH 7.4). Sections were washed three times in PBS and incubated overnight at 4°C in the primary antibody at a concentration of 1∶5,000 in PBS-T (0.2% Triton X-100 in PBS). The primary antibody used was affinity column purified rabbit anti-white-crowned sparrow GnIH (SIKPFSNLPLRF-NH_2_) antibody (code: PAC 123/124, G.E.B.). The next day, three subsequent washes in PBS were followed by incubation in biotinylated goat anti-rabbit IgG (1∶250 in PBS-T) for 1 h. After washing the sections in PBS-T three times, they were incubated for 1 h in avidin-biotin complex (ABC; Vectastain Elite Kit, Vector Laboratories, Burlingame, CA) in PBS-T. The resulting complex was visualized using 0.03% 3,3 diaminobenzidine (DAB) after washing the sections three times in PBS-T. After the structures of human GnIH peptides were indentified, the specificity of the primary antibody was assessed by adsorption tests of the antibody with 1×10^−6^ M synthetic human RFRP-1 (MPHSFANLPLRF-NH_2_), human RFRP-3 (VPNLPQRF-NH_2_), human NPAF (AGEGLNSQFWSLAAPQRF-NH_2_) and kisspeptin (YNWNSFGLRF-NH_2_). Incubation of the avian GnIH antibody with 1×10^−6^ M synthetic human RFRP-1 and human RFRP-3 completely inhibited the staining. However, the staining was not inhibited by incubating the GnIH antibody with 1×10^−6^ M kisspeptin or human NPAF.

### Double-labeling immunocytochemistry of GnIH and GnRH

To determine the relative neuroanatomical distribution of GnIH and GnRH peptides, double-labeling immunocytochemistry of GnIH and GnRH neurons was performed with slight modifications of our previous method [19]. First, sections were fixed in 4% PFA for 30 min, and after washing the sections with PBS three times they were incubated in 0.3% H_2_O_2_ in absolute methanol for 20 min to inactivate endogenous peroxidase activity. After washing the sections in PBS three times and immersing the sections in 2% normal goat serum in PBS-T, sections were incubated overnight at 4°C in the white-crowned sparrow GnIH antibody in PBS-T at a concentration of 1∶5000. The next day, three subsequent washes in PBS were followed by incubation for 1 h in biotinylated goat anti-rabbit IgG (1∶250 in PBS-T). Sections were then incubated for 1 h in ABC, and the resulting complex was visualized using Vector VIP (Vector Laboratories). The primary antibody used to label GnRH neurons was rabbit anti-GnRH (HU60H; kindly donated by Dr. H. Urbanski) at a concentration of 1∶5000 in PBS-T. The HU60 (bleed H) antiserum was generated against mammalian GnRH-I (pEHWSYGLRPG-NH_2_). HU60 equally recognizes mammalian GnRH-I, avian GnRH-I (pEHWSYGLQPG-NH_2_) and salmon GnRH (pEHWSYGWLPG-NH_2_). On the other hand, the antiserum shows no cross-reactivity with GnRH-free acid or GnRH fragments as well as other neuropeptides, including growth hormone-releasing hormone, oxytocin, somatostatin, thyrotropin-releasing hormone and vasoactive intestinal peptide [41]. The next day, three subsequent washes in PBS were followed by incubation for 1 h in biotinylated goat anti-rabbit IgG. Sections were then incubated for 1 h in ABC, and the resulting complex was visualized using DAB. Thus GnIH fibers were labeled in purple by Vector VIP, whereas GnRH neurons were labeled in brown by DAB.

### Immunoaffinity purification and mass spectrometry of human GnIH peptide

To identify the structure of the mature human GnIH peptide, we first collected GnIH-ir material from brain extract as follows, using an antiserum raised against quail GnIH [9]. Five frozen human hypothalami (total weight 5.8 g) were boiled and homogenized in 5% acetic acid, as described previously [42]. The homogenate was centrifuged at 10,000 g for 30 min at 4°C, and the supernatant was collected. The collected supernatant was passed through a disposable C-18 cartridge column (Mega Bond-Elut; Varian, Harbor, CA, USA). The retained material eluted with 60% methanol was concentrated by using an evaporator and loaded onto an immunoaffinity column. The affinity chromatography was carried out as previously described [43]. The antiserum against quail GnIH was conjugated to cyanogen bromide-activated Sepharose 4B (Amersham Pharmacia Biotech, Uppsala, Sweden) as an affinity ligand. The concentrated material was applied to the immunoaffinity column at 4°C and the adsorbed materials were eluted with 0.3 M acetic acid containing 0.1% 2-mercaptoethanol. The eluted fractions were again concentrated and subjected to a reversed-phase HPLC column (ODS-80TM; Tosoh, Tokyo, Japan) with a linear gradient of 10–50% acetonitrile containing 0.1% trifluoroacetic acid at a flow rate of 0.5 ml/min, and fractionated every 2 min for 100 min. The immunoreactive fraction was assayed by a dot immunoblot assay and the molecular mass of the material was analyzed by matrix-assisted laser desorption/ionization-time-of-flight-mass spectrometry (MALDI-TOF MS; AXIMA-CFR-plus, Shimadzu, Kyoto, Japan). MPHSFANLPLRF-NH_2_ (human RFRP-1) and VPNLPQRF-NH_2_ (human RFRP-3) were predicted as the mature peptides from the deduced amino acid sequence of the human genome sequence. Accordingly, the peptide was synthesized by using a peptide synthesizer (PSSM-8; Shimadzu) and the molecular behavior of the synthetic and native peptides was compared by MALDI-TOF MS. Furthermore, fragmentation of the synthetic and native peptides were analyzed by tandem MS.

### RT-PCR and DNA sequencing of human RFRP receptor cDNA

One human hypothalamus and one human pituitary were used for the identification of a cDNA encoding human GPR147. Total RNA (including rRNA and mRNA) was isolated by using TRIZOL (Invitrogen, Carlsbad, CA). Total RNA was reverse transcribed using oligo(deoxythymidine)15 primer (Promega, Madison, WI) and reverse transcriptase (M-MLV Reverse Transcriptase; Invitrogen). Partial human GPR147 cDNA was amplified by PCR using various primers based on human GPR147 cDNA sequence (NM_022146). Combinations of forward primer 1 (F1: 5′-ATGTTCATCCTCAACCTGGCTGTC-3′), forward primer 2 (F2: 5′-TTGTGGACAACCTCATCACTGGGT-3′), forward primer 3 (F3: 5′-TTTTCACACTGGTGGCCATTGCTG-3′) against a reverse primer (R1: 5′-ATGCGGGCGTACATGACCACGATGA-3′) produced PCR products. All PCR amplifications were performed in a reaction mixture containing Taq polymerase (TaKaRa Ex TaqTM; Takara Bio Inc., Shiga, Japan). PCR products were sub-cloned into a pGEM-T Easy vector (Promega) and the DNA inserts of the positive clones were amplified by PCR with universal M13 primers. Amplified DNA was sequenced at the UC Berkeley DNA sequencing facility (Berkeley, CA) using 3730xl DNA Analyzer (Applied Biosystems, Foster City, CA).

### *In situ* hybridization for human GnIH receptor, GPR147, mRNA and double staining with LH ICC

Coronal sections at 40 µm thickness of four frozen human pituitaries were collected on a cryostat at −20°C for histological studies. *In situ* hybridization was carried out with slight modifications of our previous method [36] using a digoxigenin (DIG)-labeled antisense RNA probe. The DIG-labeled antisense RNA probe was produced using a standard RNA labeling kit (Roche Diagnostics) by using partial GPR147 cDNA (nt 302–716 in NM_022146) as a template. Defrosted sections were first fixed in 4% paraformaldehyde (PFA) for 30 min. After washing the sections three times in PBS, they were incubated in 1 µg/ml proteinase K (Sigma-Aldrich, Saint Louis, MO) in PBS at 37°C for 30 min. Sections were fixed again in 4% PFA for 10 min, and then treated with 0.2 N HCl for 10 min after rinsing the sections in DEPC. The sections were again rinsed in DEPC twice and pre-incubated in 50% deionized formamide in 5X SSC (Roche Diagnostics) before the hybridization. Hybridization was carried out overnight at 50°C in 50% deionized formamide, 50% hybridization solution (2X concentrate, buffered with SSC, Sigma-Aldrich) at the probe concentrations of 200 ng/ml. After the hybridization, the sections were washed twice in 2X SCC in 50% formamide, and twice in 1X SSC in 50% formamide for 15 min each. After rinsing the sections in PBS, they were incubated with alkaline phosphatase-labeled sheep anti-DIG antibody (Roche Diagnostics) in 1.5% DIG blocking reagent (Roche Diagnostics) in PBS. After rinsing the sections three times in PBS and once in alkaline phosphate buffer (pH 9.5), the immunoreactive product was visualized by immersing the sections in a substrate solution (nitroblue tetrazolium/5-bromo-4-chloro-3-indolyl phosphate stock solution; Roche Diagnostics) in alkaline phosphate buffer. Control for specificity of *in situ* hybridization was performed by using a DIG-labeled sense RNA probe, the sequence of which was complementary to the antisense probe.

Immunocytochemical analyses of LH were further conducted on the same sections previously labelled by i*n situ* hybridization for human GPR147 mRNA. After labeling human GPR147 mRNA, the sections were washes in PBS three times and incubated overnight at 4°C in a rabbit polyclonal anti-human LH antiserum, (Affinity bioreagents, Golden, CO). The next day, after three washes in PBS, the sections were incubated with goat anti-rabbit IgG conjugated to Alexa Fluor 568 (Molecular Probes Inc., Eugene, OR).

### Image processing

Microscopic images were acquired digitally on an Axio Imager, A1 microscope (Carl Zeiss AG, Gottingen, Germany) with an AxioCam MRc5 digital camera (Carl Zeiss AG) using AxioVision Rel. 4.5 software package (Carl Zeiss AG). Fluorescent images were excited by using HBO 100 microscope illuminating system (Carl Zeiss AG). The brightness, contrast, sharpness and the size of the images were adjusted using Adobe Photoshop CS2 (Adobe Systems, San Jose, CA).
